# Molecular xenomonitoring of *Dirofilaria immitis* and *Dirofilaria repens* in mosquitoes from north-eastern Italy by real-time PCR coupled with melting curve analysis

**DOI:** 10.1186/1756-3305-5-76

**Published:** 2012-04-20

**Authors:** Maria Stefania Latrofa, Fabrizio Montarsi, Silvia Ciocchetta, Giada Annoscia, Filipe Dantas-Torres, Silvia Ravagnan, Gioia Capelli, Domenico Otranto

**Affiliations:** 1 Dipartimento di Sanità Pubblica e Zootecnia, Università degli Studi di Bari, Strada Provinciale per Casamassima km 3, 70010, Valenzano, Bari, Italy; 2 Istituto Zooprofilattico Sperimentale delle Venezie, Laboratory of Parasitology, Viale dell’Università 10, Legnaro, 35020, Italy; 3 Departamento de Imunologia, Centro de Pesquisas Aggeu Magalhães (Fiocruz-PE), 50670-420, Recife, Pernambuco, Brazil

**Keywords:** *Dirofilaria immitis*, *Dirofilaria repens*, Real-time PCR, Mosquitoes, Vector, Surveillance

## Abstract

**Background:**

*Dirofilaria immitis* and *Dirofilaria repens* are transmitted by bloodsucking culicid mosquitoes belonging to *Culex, Aedes, Ochlerotatus*, *Anopheles* and *Mansonia* genera.

The detection of filarioids in mosquitoes for assessing distribution of vectors and/or of pathogens in a given area (also known as “xenomonitoring”), when based on individual dissection of wild-caught female mosquitoes is time consuming and hardly applicable in large epidemiological surveys.

Our study aimed to evaluate the recently developed duplex real-time PCR for screening large number of culicids and to assess their positivity for *D. immitis* and *D. repens* in an area where both species are endemic.

**Methods:**

A duplex real-time PCR was used to detect and differentiate *D. immitis* and *D. repens* in mosquitoes collected in six provinces of the Veneto region using 43 carbon dioxide-baited traps under the frame of an entomological surveillance program to monitor the vectors of West Nile disease. From early May till October 2010, unfed female mosquitoes (n = 40,892) were captured in 20 selected sites.

**Results:**

Mosquitoes identified as *Culex pipiens*, *Ochlerotatus caspius*, *Aedes vexans* and *Culex modestus* were grouped into 995 pools according to species, day and site of collection (from minimum of 1 to maximum of 57). Out of 955 pools, 23 (2.41 %) scored positive for *Dirofilaria* spp. of which, 21 (2.2 %) for *D. immitis* and two (0.21 %) for *D. repens*. An overall Estimated Rate of Infection (ERI) of 0.06 % was recorded, being higher in *Och. caspius* and *Ae. vexans* (i.e., 0.18 % and 0.14 %, respectively). At least one mosquito pool was positive for *Dirofilaria* spp. in each province with the highest ERI recorded in Vicenza and Padova provinces (i.e., 0.42% and 0.16 %, respectively). Mosquitoes collected in all provinces were positive for *D. immitis* whereas, only two (i.e., Padova and Rovigo) provinces scored positive for *D. repens*. All mosquito species, except for *Cx. modestus*, were positive for *D. immitis,* whereas *D. repens* was only found in *Cx. pipiens*.

**Conclusions:**

The results suggest that both *Dirofilaria* species are endemic and may occur in sympatry in the examined area. The molecular approach herein used represents a powerful tool for surveillance programs of *D. immitis* and *D. repens* in the culicid vectors towards a better understanding of the epidemiology of the infections they cause and their seasonal transmission patterns.

## Background

*Dirofilaria immitis* and *Dirofilaria repens* (Spirurida, Onchocercidae) are transmitted by bloodsucking culicid mosquitoes belonging to *Culex, Aedes, OchlerotatusAnopheles* and *Mansonia* genera [1-5]. *Dirofilaria immitis* causes severe cardiopulmonary disease in dogs and it is of major veterinary importance compared to *D. repens*, which causes a low pathogenic subcutaneous infestation. Nonetheless, both filarial nematodes are of zoonotic concern worldwide being agents of human dirofilariosis [6-13].

In Europe, canine filariosis caused by *D. immitis* has been diagnosed for a long time mostly in southern regions [14], with the highest endemic area (i.e., prevalence rates as high as 80 %) along the Po River Valley of northern Italy [15,16]. Although *D. repens* remains the species most common in central and southern regions of Italy [17,18], recent reports have suggested that a change in the distribution of this parasite is occurring throughout the Italian territory [19]. Indeed, over the last decades, a high number of cases of canine dirofilariosis caused by *D. immitis* and *D. repens* occurred in areas previously regarded as non-endemic, as a consequence of the occurrence of simultaneous infections of the two species in both animal and vector populations [19-21]. In spite of the large amount of information available on the distribution of canine dirofilariosis in Europe [20], field data on vector species of *D. immitis* and *D. repens* and on the vector infection rate are exiguous [22,23].

The detection of filarioids in mosquitoes for assessing distribution of vectors and/or of pathogens in a given area (also known as “xenomonitoring”), when based on individual dissection of wild-caught female mosquitoes is time consuming and hardly applicable in large epidemiological surveys [24]. Furthermore, the morphological identification of the retrieved larval stages of *Dirofilaria* spp. is challenging and requires specialised parasitological skills, impairing a reliable, prompt diagnosis [10,25]. Over the past decades, several molecular PCR-based assays have been shown to provide rapid, sensitive, and species-specific methods for the detection and delineation of *D. immitis* and *D. repens* DNA in invertebrate hosts [22,23,26-30]. Some molecular tools have been applied especially for the entomological monitoring of human filariasis in endemic areas [24,31-33].

Nevertheless, none of these methods were used on a large scale due to inherent limitations (i.e., double species specific PCRs, low sensitivity). Recently, a sensitive SsoFast™ EvaGreen® based duplex real-time polymerase chain reaction (dqPCR) assay coupled with melting-curve analysis targeting on partial cytochrome *c* oxidase 1 (*cox*1) mitochondrial DNA and on second internal transcribed spacer (ITS-2) of nuclear ribosomal DNA [34] showed its powerfulness in sensitivity and specificity for the detection of small amounts of *D. immitis* and *D. repens* genomic DNA from dog blood and mosquito vectors.

The aims of this study were: to evaluate (i) the positivity of field collected culicids for *D. immitis* and *D. repens* in an area of north-eastern Italy (Veneto region) where both species are endemic; (ii) the usefulness of the recently developed dqPCR for screening large numbers of culicids; and (iii) the association among mosquito species captured and their positivity for *D. immitis* and *D. repens*.

## Methods

### Sampling area and mosquitoes collection

**Figure 1:**
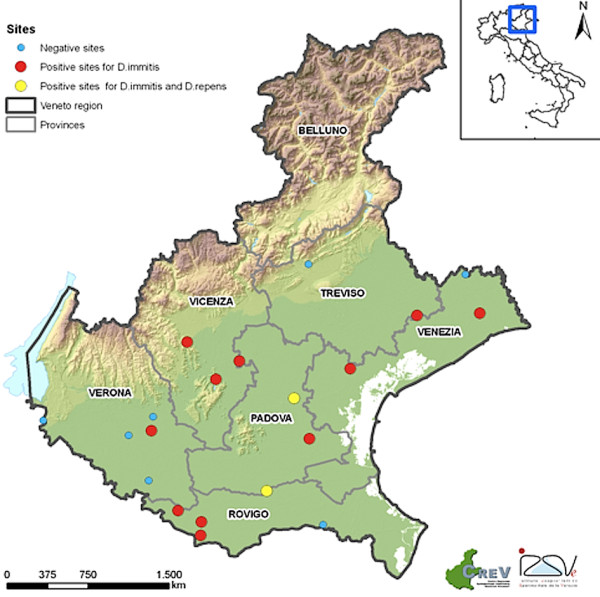
**Map of Veneto region.** Provinces and sites of mosquito collection are reported (see legend).

From May to October 2010, 43 carbon dioxide-baited traps were placed for the entomological monitoring of West Nile virus, introduced in this area since 2008 [35], in six provinces of Veneto region (north-eastern Italy). Samples herein examined came from 20 sites (Figure 1) where one or more dogs were present in the surroundings of the trap, hence restricting the survey to areas where culicid species were effectively attracted by proper hosts of *Dirofilaria* spp. The selected sites were located in rural areas, in lowland (altitude ranging from 2 to 178 m above sea level) devoted mainly to agriculture. Traps were activated every 15 days for one night from sunset to the following morning (i.e., 10.00 am). Mosquito collection was performed until two consecutive captures were negative. Females captured were visually discriminated as fed and/or unfed during identification under the stereomicroscope by size and red-brown color of the abdomen. All specimens were maintained refrigerated until being counted and identified using standard morphological keys [36].

Nine hundred and fifty-five pooled (from minimum of 1 to maximum of 57) female specimens were prepared according to species, date and site of collection. Additionally, *Cx. pipiens* naturally infested with *D. repens* (n = 2) and *D. immitis* (n = 10) were used as positive vector controls. Genomic DNA from *Aedes albopictus* and from microfilariae of both *Dirofilaria* species was mixed to obtain an artificially co-infested vector. Specimens of *Ae. albopictus* (n = 10) raised from eggs collected in the Apulia region (southern Italy), were used as vector negative control samples [34]. Genomic DNA from all mosquito samples was extracted as described in Sangioni and colleagues [37].

### Duplex real-time PCR

The duplex real-time PCR for the detection of both *D. immitis* and *D. repens* was previously assessed using two species-specific primer sets targeting *cox*1 and ITS-2 [34]. Reactions were carried out in a final volume of 20 μl, consisting of 7 μl of SsoFast^TM^ EvaGreen® Supermix (Bio-Rad Laboratories, Hercules CA, USA), 5 μl of Di-Ethyl Pyro-Carbonate (DEPC) treated pyrogen-free DNase/RNase–free water (Invitrogen, Carlsbad, CA, USA), 4 μl of template DNA (except no-template controls), 5 pmol and 100 pmol of each DI COI-F1/DI COI-R1 and Dr ITS2-F/Dr ITS2-R primer pairs, respectively. The run consisted of a hot-start at 95°C for 10 min, and 40 cycles of denaturation (95°C for 15 sec) and annealing-extension (60°C for 1 min). The melting curve was obtained by heating the product at 95°C for 20 sec, cooling it to 55°C for 20 sec and then slowly heating it at 95°C in increments of 0.5°C. The real-time PCR was performed in a CFX96^TM^ Real-Time System (Bio-Rad Laboratories, Inc., Hercules CA, USA). The increase in the fluorescent signal was registered during the extension step of the reaction and the data were analysed by the CFX Manager^TM^ Software Version 2.1 (Bio-Rad).

The specificity of the duplex real-time PCR assay was established by melting-curve analysis, and PCR products were detected by electrophoresis in 2 % ethidium bromide-stained agarose gels (Gellyphor, EuroClone, Milan, Italy), band sizes being compared with those of an appropriate molecular marker (Gene Ruler^TM^ 100-bp DNA Ladder, MBI Fermentas, Vilnius, Lithuania). Furthermore, all the real-time PCR products were purified using Ultrafree-DA columns (Millipore; Bedford, USA) and then sequenced directly using *Taq* Dye Deoxy Terminator Cycle Sequencing Kit (v.2, Applied Biosystems Inc.) in an automated sequencer (ABI-PRISM 377; Applied Biosystems Inc.), from both strands, using the same sets of primers used in the real-time PCR. All sequences generated were compared to sequences available in GenBank using Basic Local Alignment Search Tool (BLAST) [38].

### Statistical analysis

The rate of infection in mosquitoes was adjusted for pooled samples, calculating the Estimated Rate of Infection (ERI) using the following formula: ERI = 1 − (1- x/m)^1/k^[39], where *x* is the number of positive pools; *m* the number of examined pools and *k* the average number of specimens in each pool. Differences in mosquito rates of infection among species and locations were tested using Yates’ chi-squared test.

## Results

**Figure 2:**
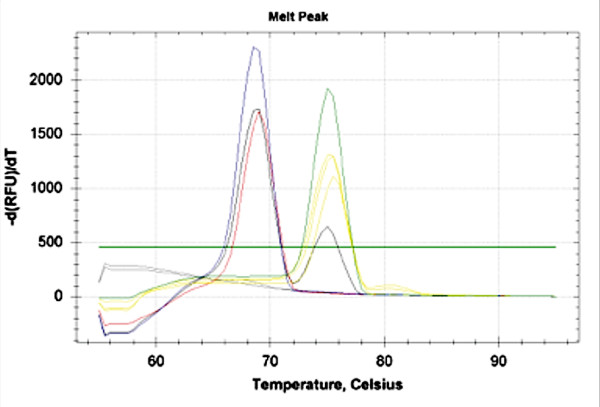
Representative melting curve analyses. Melt peak of *Dirofilaria immitis* (green), *Dirofilaria repens* (blue), and constructed positive sample with a simultaneous amplification of *cox*1 and ITS2 (black), pools of mosquito positive to *Dirofilaria immitis* (yellow) and *Dirofilaria repens* (red), respectively. Negative (*Aedes albopictus*) and No template controls (NTC) (grey).

Among the mosquito species collected, *Cx. pipiens* was the most represented (n = 37,865, 92.6 %) followed by *Och. caspius* (n = 2,264, 5.5 %), *Ae. vexans* (n = 720, 1.8 %) and *Cx. modestus* (n = 43, 0.1 %). At least one pool positive for *Dirofilaria* spp. was detected in each of the examined provinces with the highest mosquito rate of infection (ERI = 0.42 %) found in Vicenza province, where two sites were significantly more infested than all other provinces, but not Padova (Table 1). Out of the 955 mosquito pools representative for 40,892 unfed female individuals examined through the 20 sites monitored, 23 (2.41 %) scored positive for *Dirofilaria* spp. over 13 (65 %) sites (Tables 1 and 2, Figure 1). In particular, 21 pools (2.2 %) were positive for *D. immitis* and two (0.21 %) for *D. repens* by melting-curve analysis (Figure 2, Table 3). The results for positive mosquito-pool samples showed two melting peaks (i.e., mean T*m* = 75 and 69°C) corresponding to species-specific T*m* range of *D. immitis* (mean ± SD = 75.7 ± 0.3°C) and *D. repens* (mean ± SD = 70 ± 0.7°C) positive controls, respectively (Figure 2). No melting peaks generated simultaneously were displayed, except for the *Ae. albopictus* artificially co-infested vector, as well as no fluorescence signal was detected for any of the negative controls (Figure 2). *Dirofilaria immitis* was found in all mosquito species except for *Cx. modestus*, which was poorly represented, whereas *D. repens* was detected only in *Cx. pipiens* (Table 3). The overall ERI was 0.06 %, being about one mosquito infested every 1,800 (Table 2). In particular, *Och. caspius* and *Ae. vexans* showed the highest rate of infestation (0.18 % and 0.14 %, respectively). Overall, *Cx. pipiens* was about four times less infested (0.05 %) than other species and significantly less infested than *Och. caspius* (p <0.05; Table 2). However, considering single sites, *Cx. pipiens* reached the highest rate of infestation (i.e., 1.1 %) whereas the infestation rates for *D. immitis* and *D. repens* were comparable in positive sites (Table 3).

**Table 1:** **Provinces and number of sites monitored (positive and total of mosquitoes and of pools is also reported along with the estimated rate of infection (ERI) for***Dirofilaria *spp. and their statistical significance (**)).

Provinces	No. positive/ tested sites	No. mosquitoes	No. pools positive/ tested (%)	Mean no. mosquitoes per pool	ERI (%)
Padova	3/3	4652	7*/119 (5.9)	39.09	0.155
Rovigo	4/5	14478	7*/323 (2.1)	44.82	0.049^A^
Treviso	1/2	2788	1/67 (1.5)	41.61	0.036 ^b^
Venezia	2/3	6383	2/148 (1.3)	43.13	0.032 ^C^
Vicenza	2/2	1007	4/36 (11.1)	27.97	0.420 ^AbCD^
Verona	1/5	11584	2/262 (0.7)	44.21	0.017^D^
Total	13/20	40892	23/955 (2.4)	0.057	0.04-0.08

* including one positive pool for *Dirofilaria repens.*

** equal letter corresponds to significant difference (lower case = p < 0.05; upper case = p < 0.01).

**Table 2:** Number of mosquito specimens and pools tested according to their species, mean number of mosquitoes per pool and their positivity for*Dirofilaria*spp. along with the percentage of estimated rate of infection (ERI) and confidence intervals (95 % CI). Statistical significance (**)

Mosquito species	No. specimens	No. pools tested	No. pools positive	Mean no. mosquitoes per pool	ERI (%)	95 % CI
*Culex pipiens*	37865	835	18*	45.35	0.048^a^	0.03-0.07
*Ochlerotatus caspius*	2264	92	4	24.61	0.180 ^a^	0.06-0.4
*Aedes vexans*	720	25	1	28.80	0.142	0.01-0.67
*Culex modestus*	43	3	0	14.33	-	-
Total	40892	955	23	42.82	0.057	0.04-0.08

* including two positive pools for *Dirofilaria repens.*

** equal letter corresponds to significant difference.

**Table 3:** **Mosquito species and their positivity for*Dirofilaria*spp. grouped according to provinces and date of collection; mean number of mosquitoes per pool is also reported along with the percentage of estimated rate of infection (ERI) and confidence intervals (95 % CI)**

Province	Date of collection	Number of sites	Mosquito species	*Dirofilaria* species	No. specimens	No. pools positive/tested	Mean no. mosquitoes per pool	ERI*	95 % CI
PD	25/05/2010	18	*Cx. pipiens*	*D. immitis*	204	1/5	40.80	0.545	0.03-2.58
	08/06/2010	18	*Cx. pipiens*	*D. immitis*	384	1/8	48	0.278	0.01-1.32
	17/08/2010	18	*Cx. pipiens*	*D. immitis*	9	1/1	9	na	
	28/09/2010	18	*Och. caspius*	*D. immitis*	2	1/1	2	na	
	20/07/2010	12	*Cx. pipiens*	*D. repens*	160	1/4	40	0.717	0.03-3.42
	14/09/2010	12	*Cx. pipiens*	*D. immitis*	259	1/6	43.17	0.421	0.02-2.01
	05/10/2010	10	*Och. caspius*	*D. immitis*	2	1/1	2	na	
RO	11/05/2010	14	*Cx. pipiens*	*D. immitis*	3	1/1	3	na	
	08/06/2010	16	*Cx. pipiens*	*D. immitis*	862	2/18	47.89	0.246	0.04-0.77
	20/07/2010	16	*Cx. pipiens*	*D. immitis*	458	1/10	45.80	0.230	0.01-1.08
	20/07/2010	16	*Cx. pipiens*	*D. repens*	458	1/10	45.80	0.230	0.01-1.08
	06/07/2010	163	*Cx. pipiens*	*D. immitis*	507	1/10	50.70	0.208	0.01-0.98
	31/08/2010	162	*Cx. pipiens*	*D. immitis*	109	1/3	36.33	1.110	0.05-5.26
TV	01/06/2010	179	*Och. caspius*	*D. immitis*	2	1/1	2	na	
VE	15/06/2010	199	*Cx. pipiens*	*D. immitis*	450	1/9	50	0.235	0.01-1.11
	26/08/2010	4	*Ae. vexans*	*D. immitis*	8	1/1	8	na	
VI	04/05/2010	187	*Cx. pipiens*	*D. immitis*	1	1/1	1	na	
	18/05/2010	187	*Cx. pipiens*	*D. immitis*	1	1/1	1	na	
	07/09/2010	187	*Cx. pipiens*	*D. immitis*	8	1/1	8	na	
	22/09/2010	183	*Cx. pipiens*	*D. immitis*	20	1/1	20	na	
VR	13/07/2010	191	*Och. caspius*	*D. immitis*	3	1/1	3	na	
	07/09/2010	191	*Cx. pipiens*	*D. immitis*	50	1/1	50	na	

* na = not applicable.

**Figure 3:**
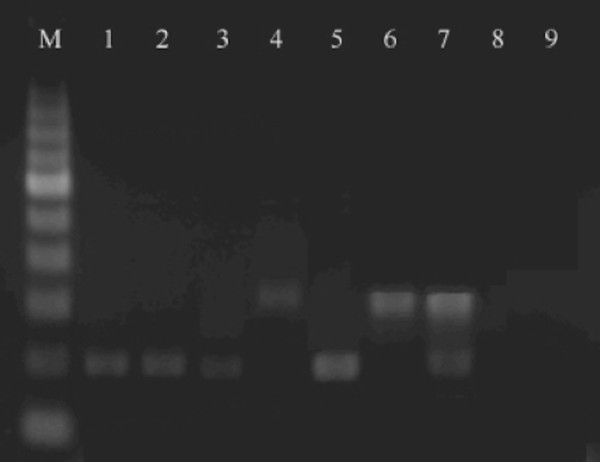
**Real-time PCR patterns on 2% ethidium bromide-stained agarose gels.** M, 100 bp DNA marker. Lane 1–3, *Dirofilaria immitis* (positive samples); Lane 4, *Dirofilaria repens* (positive samples); Lane 5–7, positive controls; Lane 8, negative control (*Aedes albopictus*); Line 9, No template control (NTC).

All real-time PCR products were confirmed by detection on 2 % ethidium bromide-stained agarose gels showing a single band of 200 and 300 bp for *D. immitis* and *D. repens*, respectively (Figure 3). The sequences derived from the amplicons matched (99-100 % identity) appropriate reference sequences of *Dirofilaria* species (accession numbers JF461464 and AY693808). Positive pools were found each month throughout the sampling period from the first week of May (start of monitoring) till October, without any apparent seasonality (Table 3).

## Discussion

This study reports data on the first survey employing a duplex real-time PCR to assess the occurrence of *D. immitis* and *D. repens* in culicids collected in a large-scale entomological monitoring programme and provides information about the relative infestation rate for both filarial nematodes in mosquito species of north-eastern Italy. Collecting mosquitoes under the frame of a regional surveillance programme for the West Nile virus vectors allowed optimizing samples and collection efforts, thus saving economic resources.

Results indicate that *Dirofilaria* spp. were evenly detected in mosquito pools throughout the Veneto region with the highest rate of infestation found in Vicenza and Padova (i.e., ERI = 0.42% and 0.16 %, respectively). In particular, while *D. immitis* was detected in mosquito pools collected in all provinces, *D. repens* was found only in Padova and Rovigo. The results above confirm the occurrence of *D. repens* in north-eastern Italy, where the species was reported more than 20 years ago [40], it also expands current knowledge on the distribution of this parasite, which was previously detected in western-regions of the country (Piedmont and Lombardy) [15,41,42]. Interestingly, the high *D. immitis* infestation rate in *Och. caspius* (i.e., overall ERI = 0.18 % and local ERI = 0.7 %) indicates the role of this species as a putative vector, as previously suggested, based on the retrieval of infective larvae at the dissection [43]. Conversely, a molecular study based on classic PCR failed to detect *D. immitis* in *Och. caspius*, indicating that this mosquito species was refractory to filarial infestation [44]. *Aedes vexans* was found positive for *D. immitis* only once in a small number of mosquitoes, corresponding to a considerable infestation rate (i.e., ERI = 0.14 %). A similar result was already reported in a study from Turkey in which this mosquito species was suggested as the main vector of *D. immitis*[45]. However, this finding needs to be confirmed in a larger collection of *Ae. vexans*, poorly represented in our study likely because of its diurnal activity. Previous studies carried out in central and northern Italy failed to detect *D. immitis* in *Ae. vexans*, both by insect dissection and classic PCR testing [44,46]. Hence, our findings might be a result of either the higher sensitivity of our PCR protocol in detecting genomic *D. immitis* DNA (detection limit of the assay as low as 2.5 pg/μl of DNA) [34] or of the effective presence of this nematode in our sampled *Ae. vexans* population. *Culex modestus* scored negative for *D. immitis* in contrast to previous reports indicating this species as a competent vector [47,48]. This result may be due to the small number of mosquitoes specimens collected, due to its host preference. Indeed, *Cx. modestus* is considered unwilling to feed on dogs [41,49-52], differently from *Och. caspius* and *Cx. pipiens*. The latter species was highly positive for *D. immits* and *D. repens* as already demonstrated by insect dissection and/or PCR processing [22,53].

Even if the present assay does not allow differentiation between infested and infective mosquitoes (abdomen and thorax-head have not been analyzed separately), data herein presented clearly indicate the usefulness of the molecular diagnosis assay for a broader field application in order to gain more information on the epidemiology of the mosquitoes acting as vectors of *Dirofilaria* spp.

Moreover, an effective surveillance system is pivotal considering the expansion of certain vectors of *Dirofilaria* spp. throughout areas previously regarded as non-endemic. This is the case of *Ae. albopictus,* a mosquito species highly adapted to many ecological niches in southern Europe [54] which, in turn, is also a more competent vector of *D. immitis* than native *Cx. pipiens* populations [23,28]. Importantly, continuous epidemiological surveillance systems need to be implemented by local authorities in order to test autochthonous vector populations as well as recently introduced potential vectors (e.g., *Aedes koreicus*) [55]. A continuous monitoring of infestation rates for mosquito vector populations is pivotal to evaluate the success of an anti-filarial campaign in endemic areas, thus providing evidence on whether drug administration in a given area can be terminated or not, in order to reduce risk for drug resistance in filarioid populations. The advantages of using a highly sensitive molecular diagnostic tool for xenomonitoring was shown to be pivotal for assessing the efficacy and progress of eradication programmes of lymphatic human filariasis based on mass drug administration [56,57].

## Conclusions

The results presented here suggest a high sensitivity and specificity of the assay herein used in field-collected samples, clearly representing an alternative to classic microscopic methods and to PCR-based assays for the xenomonitoring of *D. repens* and *D. immitis*, mainly in areas where these species are endemic and/or occur in sympatry [4,58]. Moreover, it represents a non-invasive method to assess the presence of both *Dirofilaria* species in an area. Field information on vector distribution and their rates of infestation will contribute to implementing warning among veterinarians, physicians and health authorities, also considering that human dirofilariasis is reported to be increasing in the Old World [20].

## Competing interests

The authors declare that they have no competing interests.

## Authors’ contributions

FM, SC, and SR collected and identified the mosquito specimens. MSL and GA run the real-time PCRs. GC, LMS and DO contributed in writing the first draft of the manuscript. LMS, DO, FDT and GC equally contributed with data analysis and interpretation and revision of the manuscript. All authors read and approved the final version of the manuscript.
